# Who Is at Risk for Preeclampsia? Risk Factors for Developing Initial Preeclampsia in a Subsequent Pregnancy

**DOI:** 10.3390/jcm9041103

**Published:** 2020-04-13

**Authors:** Tamar Wainstock, Ruslan Sergienko, Eyal Sheiner

**Affiliations:** 1 Department of Public Health, Faculty of Health Sciences, Ben-Gurion University of the Negev, Beer-Sheva 84105, Israel; 2 Department of Obstetrics and Gynecology, Soroka University Medical Center, Ben-Gurion University of the Negev, Beer-Sheva 84105, Israel

**Keywords:** preeclampsia, pregnancy complications, preterm birth, gestational diabetes, nested case control

## Abstract

Background: The incidence of preeclampsia, which may cause significant maternal and perinatal morbidity, has risen in recent years, therefore it is critical to identify women at risk for preeclampsia. We aimed to identify risk factors in the first pregnancy (not complicated by preeclampsia) for preeclampsia in the subsequent pregnancy. Methods: A retrospective population-based nested case-control study was conducted, including all women with first (P1) and second (P2) singleton consecutive deliveries. Women who had experienced preeclampsia in their first pregnancy were excluded. Cases were defined as women with preeclampsia in their second pregnancy, and were compared to the controls, defined as women without this diagnosis in second pregnancy. Characteristics and complications of the first pregnancy were compared between cases and controls, and multivariable regression models were used to study the association between pregnancy complications (in the first pregnancy) and preeclampsia (in the subsequent pregnancy), while adjusting for confounders. Results: A total of 40,673 women were included in the study, 1.5% of second pregnancies were diagnosed with preeclampsia (*n* = 627, i.e., Cases). Cases, as compared to controls were older in their 1st pregnancy, with longer inter-pregnancy interval, and were more likely to have the following complications in their first pregnancy: preterm delivery (15.0% vs. 7.7%), low birthweight (17.9% vs. 10.3%), perinatal mortality (3.2% vs. 1.1%), and gestational diabetes (7.0% vs. 2.7%). In the multivariable model, adjusted for maternal age, obesity and inter-pregnancy interval, either one of these first pregnancy complications were independently associated with an increased risk for preeclampsia (adjusted OR for either of first pregnancy complication =1.73; 95% CI 1.37–2.14, <0.001), and the risk was greater for each additional complication (adjusted OR for ≥2 risk factors =3.54; 95% CI 2.28–5.52, *p* < 0.001). Conclusions: Complications in first pregnancy, including preterm delivery, perinatal mortality and gestational diabetes, are risk factors for primary preeclampsia in second pregnancy. First pregnancy may serve as a window of opportunity to identify women at risk for future preeclampsia and other morbidities later in life.

## 1. Introduction

Preeclampsia, affecting approximately 3%–7% of all deliveries, is a leading cause of maternal and perinatal morbidity [1]. Incidence of initial preeclampsia in second pregnancy is ~2.0% [2]. In many countries, incidence of pregnancies complicated with preeclampsia has increased, and even doubled, in recent decades, mainly due to demographics changes over the years, such as advanced maternal age and obesity, which are considered risk factors for preeclampsia.

Besides the immediate risk preeclampsia poses to the pregnancy, the mother and the fetus, offspring prenatally exposed to preeclampsia may be at risk for long term adverse health effects [3,4,5]. Women with a history of preeclampsia are at increased risk for long-term morbidities, including chronic hypertension, diabetes and atherosclerotic morbidity such as cardiovascular and renal disease, besides preeclampsia in future pregnancies [6,7,8]. In fact, in recent years preeclampsia is recognized as a systemic disease with widespread effects, which does not end with placental delivery, as commonly accepted for many years [9].

Preeclampsia etiology usually involves abnormal placentation, as well as sub-optimal interaction between maternal factors with a healthy placenta [9,10]. While the actual cause of preeclampsia is mainly unknown, several risk factors for preeclampsia have been identified, including a history of preeclampsia, maternal obesity, and other maternal chronic health conditions including hypertension, diabetes, and chronic heart diseases [11,12,13]. Some of these factors, such as obesity and untreated chronic morbidities, can be treated before pregnancy and thus may reduce the risk.

Several treatment and prevention options have been suggested for preeclampsia, not all have been proven valuable. These interventions address the different pathogenesis possibly involved in preeclampsia. These include calcium, vitamin C and E supplementation, or Aspirin prescription, which has been suggested to efficiently prevent preeclampsia among women at high risk [14,15,16], specifically in preterm preeclampsia and if initiated early in pregnancy. Additional treatments include medications to prevent hypertension and seizures [15,17].

A better understanding of preeclampsia etiology and identification of additional women at risk, is of major importance since prevention and treatment of women before or early in pregnancy may improve both immediate and long-term maternal and offspring health. The aim of this study was to identify risk factors based on obstetrical history for primary preeclampsia in the second pregnancy.

## 2. Experimental Section

A retrospective population- based nested case- control study was conducted, including all women with two first singleton consecutive deliveries of first two consecutive pregnancies (with no pregnancies before the 1st or the 2nd pregnancies that ended in abortion), between the years 1991–2017. Only women with documented and accurate matching of parity and gravidity status, and with full medical records on both pregnancies and deliveries were included. Preeclampsia was defined based on either one of the following ICD-9 codes: 642.41; 642.42; 642.51; 642.52; 642.61; 642.62. Women with preeclampsia in the first pregnancy were excluded, as well as multiple gestations (in either pregnancy). The study was conducted at the Soroka University Medical Center (SUMC) located in the Southern region of Israel. SUMC, the sole tertiary medical center in the region, serves a population of >1 million residents, and has the country‘s largest birthing center.

Cases were defined as women with preeclampsia in their second pregnancy, and they were compared to the controls, defined as women without preeclampsia in their second pregnancy. Characteristics and complications of the first pregnancy were compared between cases and controls, using chi-square test and student *t*-tests. First pregnancy characteristics and complications that were significantly different between cases and controls were tested in the multivariable analysis. First pregnancy characteristics that were highly correlated were not included in the model together, and only one was selected based on the best fit (minimal log likelihood). Multivariable logistic regression models were used to study the association between pregnancy complications (in the first pregnancy) and preeclampsia (in the subsequent pregnancy), while adjusting for maternal age and inter-pregnancy interval (IPI). IPI was calculated as the number of years between first delivery and best estimation of first day of last menstruation period of the second pregnancy, based on clinical evaluation and first trimester sonar test. Long IPI was evaluated as a collider variable in the association between the risk factors and preeclampsia. In addition to specific pregnancy complications, a combined adverse pregnancy score was created, which summed the number of first pregnancy complications which were associated with second pregnancy preeclampsia (based on the first step analysis): preterm delivery (<37 gestational weeks), gestational diabetes and neonatal mortality. Scoring ranged between 0 = no complications; 1 = one complication, 2 = two or 3 complications. A multivariable model was then used to study whether the risk for preeclampsia in the second pregnancy increased with each additional complication. Women with no first pregnancy complications served as the reference group. The study protocol received SUMC IRB approval, and informed consent was exempt.

## 3. Results

A total of 40,673 women were included in the study (81,346 deliveries), 1.5% of second pregnancies were diagnosed with preeclampsia (*n* = 627, i.e., Cases). Their diagnoses were as follow: 5 (<0.1%) eclampsia; 111 (17.7%) severe preeclampsia; 511 (81.5%) mild preeclampsia. Yearly incidence of preeclampsia ranged between 3.0% and 0.8% during the study period.

Cases, as compared to controls were older in their first pregnancy, and were more likely to have the following complications in their first pregnancy Table 1: preterm delivery (15.0% vs. 7.7%; OR = 2.11; 95%CI 1.69–2.63, *p* < 0.01), low birthweight (17.9% vs. 10.3%; OR = 1.90; 95%CI 1.54–2.34, *p* < 0.01), perinatal mortality (3.2% vs. 1.1%; 2.85; 95%CI 1.81–4.49, *p* < 0.01), and gestational diabetes (7.0% vs. 2.7%; 2.69; 95%CI 1.97–3.67, *p* < 0.01). Inter-pregnancy interval was longer among cases than controls (2.06 ± 1.8 vs. 1.53 ± 1.5; *p* < 0.001).

While the incidence of second pregnancy preeclampsia was 1.5% in the total study population, among women with first pregnancy low birthweight the incidence was 2.7%; among women with first pregnancy preterm delivery the incidence was 3.0%; among women with first pregnancy gestational diabetes the incidence was 3.9%; and among women with first pregnancy fetal mortality the incidence was 4.2%.

**Figure 1:**
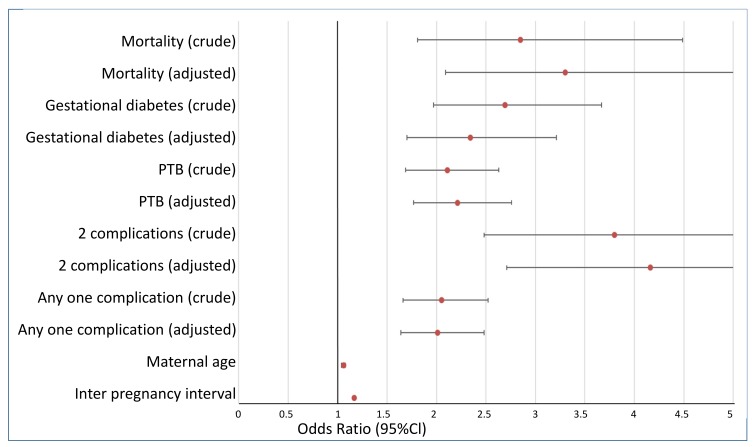
Adjusted (for maternal age and inter-pregnancy interval) and unadjusted odds ratios for the associations between first pregnancy complications and preeclampsia in subsequent pregnancy.

In separate multivariable models, while adjusting for maternal age and inter pregnancy interval, each of the following first pregnancy complications were associated with an increased risk for second pregnancy preeclampsia: low birthweight; preterm delivery; gestational diabetes and fetal mortality (results presented in Figure 1). A significant correlation was found between perinatal mortality and preterm deliveries (r, spearman = 0.25, *p* < 0.001) and between low birthweight and preterm birth (r, spearman = 0.53, *p* < 0.001).

Table 2 presents results for the combined adverse pregnancy score, in which women who scored 1 or 2 in the combined adverse pregnancy score were compared to women scoring 0. The combined score included the following complications: preterm delivery, gestational diabetes and fetal mortality. Low birthweight was not included in the analysis since it correlated with preterm delivery. The model adjusted for maternal age >35, obesity and categories of inter-pregnancy interval. Having a history of either one of the first pregnancy complications, was independently associated with an increased risk for preeclampsia (adjusted OR for a single risk factor = 2.12; 95% CI 1.72–2.61, *p* < 0.001), and the risk was higher when having 2 or more complication (adjusted OR = 4.82; 3.12–7.44, *p* < 0.001).

## 4. Discussion

It is well established that women with a history of preeclampsia are at increased risk for its recurrence. In the present study, additional women have been identified during first pregnancy, to be at increased risk for subsequent preeclampsia.

As expected, obese women or with a diagnosis of gestational diabetes mellitus in first pregnancy, although not diagnosed with preeclampsia in their first pregnancy, were at an increased risk for preeclampsia in their second pregnancy. This association was independent from their age and inter- pregnancy interval, and suggest these women had sub-clinical, un-diagnosed preeclampsia in their first pregnancy. Women diagnosed with gestational diabetes mellitus in first pregnancy may have been diagnosed with diabetes following first delivery, and were therefore at an increased risk for preeclampsia in the subsequent pregnancy [11].

The association between preeclampsia and having a history of preterm delivery has been previously reported [18]. Preterm deliveries, perinatal mortality or low birthweight all may share common pathophysiology, involving utero-placental hypo-perfusion, which may be expressed as preeclampsia in the subsequent pregnancy [17,19,20]. In the last decade, studies have found several biomarkers during early pregnancy attempting to predict preeclampsia, including antiangiogenic sFlt-1 (soluble fms-like tyrosine kinase-1) and low levels of proangiogenic PlGF (placenta growth factor protein), the ratio between sFlt-1/PlGF, circulating RNA and cystatin-c [13,21,22,23,24,25]. Still, screening for preeclampsia biomarkers is not routinely practiced, and clinical characteristics, including maternal background health and reproductive history, are mainly used to identify women at increased risk for this complication [17].

Regarding the strengths and limitations of this study, as in all studies, and specifically those of a retrospective nature, it is expected that not all confounding variables were accounted for, and in this study data regarding changes in socioeconomic or environmental characteristics between the two pregnancies, which have been associated with preeclampsia risk, were unavailable. Still, the major health factors were available and accounted for, as well as the inter-pregnancy interval. It can be assumed, that changes in socioeconomic or environmental characteristics between the pregnancies are strongly correlated with inter-pregnancy interval and would be minimal with shorter inter-pregnancy interval and vice versa. Data regarding the timing of preeclampsia onset (early or late) was also unavailable, and so differences in risk factors between the two types of preeclampsia could not be addressed. Future studies are recommended to address these possible differences, as well as the the effectiveness of aspirin administration for women with previous history of gestational diabetes, preterm delivery or perinatal mortality, and even more so, among women with more than one of these complications, which were found in the current study to be associated with preeclampsia risk.

## 5. Conclusions

The pregnancy period, in which otherwise healthy women are under relatively intense medical surveillance, has been recognized as a window of opportunity to identify women at risk for future morbidities. Certain pregnancy complications including preeclampsia, can be prevented and the long term adverse health affect, for both the mother and the offspring, possibly modified. It is therefore important to identify women which can benefit from treatment and prevention strategies, aiming to reduce incidence of primary preeclampsia in second pregnancy. Such intervention will can also reduce costs for health systems [22]. While a history of preeclampsia is a risk for its recurrence, our finding suggest women should also be asked regarding additional first pregnancy complications, as other complications are also associated with increased preeclampsia risk.

## Figures and Tables

**Table 1:** First pregnancy characteristics by cases and controls.

Characteristics	Cases (Preeclampsia in 2nd Pregnancy) *N* = 627 (1.5%)	Controls (No Preeclampsia in 2nd Pregnancy) *N* = 40,046 (98.5%)	OR; 95%CI	*p*-Value
Maternal age (mean± SD)	26.74 ± 4.8	25.47 ± 4.5		<0.001
Birthweight (mean± SD)	2,940 ± 647	3,061 ± 505		<0.001
Gestational age (mean± SD)	38.4 ± 2.9	39.01 ± 2.1		<0.001
Cesarean delivery, *n* (%)	102 (16.3)	4,711 (11.8)	1.46; 1.18–1.81	0.001
Preterm delivery (<37 gestational weeks), *n* (%)	94 (15.0)	3,091 (7.7)	2.11; 1.69–2.63,	<0.001
Low birthweight (<2,500 g.), *n* (%)	112 (17.9)	4,113 (10.3)	1.90; 1.54–2.34	<0.001
Intrauterine growth restriction, *n* (%)	29 (4.6)	1,166 (2.9)	1.62; 1.11–2.36	0.013
Small for gestational age (<5th percentile), *n* (%)	60 (9.6)	3,216 (8.0)	1.21; 0.93–1.59	0.16
Obesity, *n* (%)	14 (2.2)	260 (0.6)	3.49; 2.03–6.02	<0.001
Perinatal mortality, *n* (%)	20 (3.2)	458 (1.1)	2.85; 1.81–4.49	<0.001
Gestational diabetes, *n* (%)	44 (7.0)	1,094 (2.7)	2.69; 1.97–3.67	0.001
Placental abruption, *n* (%)	5 (0.8)	196 (0.5)	1.63; 0.67–3.98	0.24
Inter pregnancy interval, years (mean ± SD)	2.06 ± 1.8	1.53 ± 1.5		<0.001

**Table 2:** Multivariable analysis for the association between first pregnancy complications and perinatal mortality risk in second pregnancy.

Variable	Adjusted Odds Ratio; 95%CI	*p*-Value
No complications in previous pregnancy *	1 (reference)	
1 complication	2.08; 1.69–2.57	<0.001
≥2 complications	4.61; 2.99–7.10	<0.001
Maternal age (years)	1.04; 1.02–1.06	<0.001
Inter pregnancy interval	1.11; 1.07–1.16	<0.001

* the following complications were included: preterm delivery, gestational diabetes and neonatal mortality.
